# The "Normyl Treatment" of Alcoholism

**Published:** 1912-05-11

**Authors:** 


### THE "NORMYL TREATMENT" OF ALCOHOLISM.

Of all technical things, medical therapeutics is the one upon which the uninitiated venture most readily. That the patient is the only judge of the results of treatment, and that doctors are a body of men whom self-interest and pride constantly prevent from taking valuable hints from others, are two propositions which most laymen hold firmly to in their hearts. The fallacies of the one, and the amiable assumption in the other, have been often exposed; they need not detain us here. Anxiety to be in the front of a successful new departure, and the joy of confounding experts, and overmuch leisure, and the sense of possession of money and influence of which some striking use should be made, are contributory causes in bringing to light so many amateur pharmacologists. The medical profession is constantly being taught its business by Ladies Bountiful of both sexes. Whether it be the nostrum of a Parisian chemist, or of a rustic herbalist, many persons of position will be found to go over the heads of the proper judges and force on the notice of the public some dubious preparation or other. We do not say this has been the case with the one named above, but, all the same, it would seem well now to draw the attention of all and sundry to the fact that in a recent number of the *British Medical Journal* there appears an analysis of this remedy for drunkenness, and that it is found to contain a good deal of alcohol.

This is a remarkable finding, not only on the face of it, but for the following reasons. It is well known that the depravity of human nature has found a means of money-getting in advertising "cures" for drug habits, which "cures" really consist largely of the drug itself. Thus there are opium "cures" strongly fortified with morphine, and so on. Last week's *Lancet* castigates one of these iniquitous secret "cures" for morphinism. Obviously the latter state of the victim taking such treatment differs from his first one only in this, that whereas to begin with he buys the drug in the open market, after his attempt at reform his custom goes to a particular individual. Again, let us immediately add that we do not impute these motives to promoters of the "Normyl treatment"; in fact, we think any such supposition most unlikely. It is possible, although what was analysed is stated to have been the latter half of medicine for a twenty-four days' course, that alcohol was present in each day's quantum in successively diminishing doses, with the presumed object, which certain directions suggest, of weaning the patient by degrees. This must remain uncertain, because the quantity in each phial was too small for separate full analysis. Strychnine was undoubtedly present, and also in all probability cocculus indicus, with an orange flavouring. So much for this "cure for alcohol and drug addictions," some of which the patient had to take every hour while awake.

Now, even putting, as we do, the most favourable interpretation—the presence of cocculus indicus was not indubitable—on the scientific data opportunely furnished by our contemporary, is there anything at all in this cure to warrant fuss being made, meetings held, and an organisation set up? Have our medical officers of inebriate homes and retreats, or short of them a family physician backed up by loving friends, the very slightest to learn from the "Normyl" system? Here we have at best indications of the tritest measures, as is so often the case with secret remedies. Those who are not scientifically educated enough to have learnt the all sufficient reasons against secrecy must put up with the *exposé* when it comes, as it always, sooner or later, will come. The waste of resources and of public attention is obvious. It will be a glad day when responsible persons learn that the medical profession is as much the best judge of therapeutic means, as a fully qualified engineer is of how to go about building a bridge. Priming a man with drugs in pharmaceutical doses will not break his neck, it is true, but at best it is hardly a rational use of time.

